# From Mind to Mouth: Event Related Potentials of Sentence Production in Classic Galactosemia

**DOI:** 10.1371/journal.pone.0052826

**Published:** 2012-12-26

**Authors:** Inge Timmers, Bernadette M. Jansma, M. Estela Rubio-Gozalbo

**Affiliations:** 1 Department of Pediatrics, Maastricht University Medical Center, Maastricht, The Netherlands; 2 Department of Cognitive Neuroscience, Maastricht University, Maastricht, The Netherlands; 3 Maastricht Brain Imaging Center, Maastricht University, Maastricht, The Netherlands; 4 Laboratory of Genetic Metabolic Diseases, Maastricht University Medical Centre, Maastricht, The Netherlands; University of Leicester, United Kingdom

## Abstract

Patients with classic galactosemia, an inborn error of metabolism, have speech and language production impairments. Past research primarily focused on speech (motor) problems, but these cannot solely explain the language impairments. Which specific deficits contribute to the impairments in language production is not yet known. Deficits in semantic and syntactic planning are plausible and require further investigation. In the present study, we examined syntactic encoding while patients and matched controls overtly described scenes of moving objects using either separate words (minimal syntactic planning) or sentences (sentence-level syntactic planning). The design of the paradigm also allowed tapping into local noun phrase- and more global sentence-level syntactic planning. Simultaneously, we recorded event-related potentials (ERPs). The patients needed more time to prepare and finish the utterances and made more errors. The patient ERPs had a very similar morphology to that of healthy controls, indicating overall comparable neural processing. Most importantly, the ERPs diverged from those of controls in several functionally informative time windows, ranging from very early (90–150 ms post scene onset) to relatively late (1820–2020 ms post scene onset). These time windows can be associated with different linguistic encoding stages. The ERP results form the first neuroscientific evidence for language production impairments in patients with galactosemia in lexical and syntactic planning stages, i.e., prior to the linguistic output phase. These findings hence shed new light on the language impairments in this disease.

## Introduction

Patients with classic galactosemia, an inborn error of galactose metabolism, have speech and language production impairments, whereas comprehension is relatively preserved [1], [2]. Such impairments can be burdensome to patients as they might hamper communication and hence social interactions. Nevertheless, underlying language processing components and neural correlates of these impairments are poorly understood.

In classic galactosemia, there is a deficiency of the enzyme activity that converts galactose-1-phosphate (Gal-1-P) into UDP-galactose (i.e., the galactose-1-phophate uridyl transferase [GALT] enzyme). This is due to mutations in the GALT gene, located on the short arm of chromosome 9. A galactose-restricted diet resolves the neonatal toxic symptoms, but cannot prevent the emergence of cognitive difficulties such as lowered intelligence, memory problems, slower general information processing and impaired speech and language production [1], [3]–[10], while receptive language or comprehension is relatively preserved [1]. Voice and motor speech disorders (e.g., childhood apraxia of speech or dysarthria) have been reported [9], [11]–[14] as well as problems with word retrieval, grammar and vocabulary (the latter impairments are related to the planning of a message and not with the verbal output of a message) [2], [5], [6]. Although patients can experience a broad spectrum of cognitive impairments, the speech and language impairments cannot be solely explained by lower cognitive abilities in general [1], [2], [6] (e.g., some patients with low intelligence have no language impairments, while others with average intelligence have language impairments [1]). Hitherto, the main focus of research, diagnosis and treatment has been on speech (output) difficulties (e.g., on voice disorders or childhood apraxia of speech [9], [11]–[13]). However, speech (output) impairments cannot solely explain the language impairments. Language production is a complex process comprising multiple processing stages prior to the output stage [15]–[17]. In galactosemia, nonetheless, it has never been studied how language production is affected. In this study, we took a cognitive point of view, examining language production using psycholinguistic models. In the remainder of this manuscript we will refer to ‘language production’ as specified in the field of linguistics and cognitive neuroscience, namely describing the cognitive phases that are involved in planning a message prior to the articulation.

Psycholinguistic models of language production suggest cognitive stages in which relevant language information is planned over time. First, an intended message has to be transferred into a conceptual/semantic representation. Appropriate lexical entries are selected and retrieved as well as the corresponding grammatical and syntactic information. Structural syntactic frames are constructed and assembled or filled in producing a well-formed utterance. Finally, the message is encoded and articulated [15], [18]. The language production process has been investigated in many picture naming experiments using reaction times (see [19] for a review) and event related potentials (ERPs, derivatives of the electroencephalogram [20]). This way, sensitive time windows have been suggested for the language production stages. It has been shown that conceptual information is activated around 120 ms after stimulus onset [21], followed by semantic processing. This is followed by syntactic encoding approximately 90 ms later, serving as input to phonological encoding after another 40 ms. The processes are not fully serial but might overlap in time, suggesting cascading information flow over time [16], [22]–[24]. Each of the planning steps can be linked to specific brain areas within a cortical network [16], [25], [26]. Ignoring other potentially relevant factors for a moment, any type of impairment might therefore be directly related to dysfunction within this network. Lesions within specific areas may affect production and comprehension separately [27], [28], whereas disruptions of connectivity between areas may delay or disturb language processing [29]. Few imaging studies have been conducted in galactosemia, observing anatomical brain abnormalities, such as white matter abnormalities, cerebral and cerebellar atrophy [30], , but it remains uncertain whether specific areas or networks might be particularly affected.

A screening of our patient cohort's medical files suggested a syntactic deficit in the galactosemia patients as their utterances were described as short, simple and frequently as syntactically incorrect. Necessary steps in syntactic encoding are identification and activation of grammatical information associated with the concepts (e.g., whether it is a noun or adjective; lexical selection), the assignment of syntactic relations or grammatical functions to each word (e.g., subject versus object; function assignment), inflection of words (e.g., -*s* for plural, -*ed* for past tense) and assembly of words into so called syntactic structural frames, i.e., syntactic plans (constituent assembly) [15], [18]. It deduces that in syntactic planning more local phrasal-level planning (first steps described) can be distinguished from more global sentence-level planning (assembly into a frame and utterance). Especially in multi-word utterances, it is believed that the scope of planning is incremental such that the utterance can be initiated as soon as certain elements are available [15], [17], [32], [33]. The amount of advance planning is suggested to be in terms of functional phrases [33], but is also dependent on the cognitive load of the utterance and the cognitive capacity of the speaker [15], [34], [35]. In healthy controls, syntactic processing has been studied in the context of syntactic anomalies or syntactic complexity during comprehension (P600 and left anterior negativity [LAN] ERP components) [36], [37]. In terms of brain areas, syntactic encoding and sentence processing have been related to the left inferior frontal gyrus (LIFG, encompassing Broca's area) [25], [38]–[40]. In comprehension research, studies assume that the LIFG is retrieving and integrating lexical information from long term memory, most likely from left temporal areas [36], [41]–[43]. A similar process can be assumed for speech planning in which concepts have to be integrated into proper syntactic and phonological frames (see [39], for first empirical indications using intracranial electrophysiology).

In the present study, we aimed to investigate whether patients with galactosemia have impairments in sentence production by recording high temporal resolution ERPs during a language task. This method allowed us to track the neural activity related to the entire language planning process from the intention to speak onwards, across sensitive time windows. Comparing the patients' ERP (i.e., morphology of the wave, amplitude and latency of components) with that of healthy controls gives us an indication on whether syntactic encoding is intact, delayed, or malfunctioning at a millisecond time resolution. An experimental paradigm was used that elicits overt utterances in response to an animated scene in a relatively natural manner. Through different instructions, the reports of the scene varied in syntactic complexity [38], , allowing us to study syntactic effects within the ERP. The content of the scenes differed from trial to trial (i.e., the geometrical figure, colour of the figures and verb) and not all information was available from the scene onset (i.e., the verb; the actor could either ‘*bump into*’ or ‘*fly towards*’ the other figure; both scene variations start visually identical, and diverge at a certain point). The participants therefore could not anticipate the action of the figure, ensuring active generation of the utterances (instead of only automated processes). Further, it allowed us to tap into both early local phrasal-level planning of noun phrases (starting immediately after scene onset, associated with initiation of planning the first elements of the utterance that are already available: the first nouns and corresponding adjectives) and on later global sentence-level planning (when all relevant information is at hand, including the verb; adding the construction of the utterance). Time windows of any deviations, relative to the visual stimulation, give information on whether differences are related to early conceptual, early local syntactic, or rather late global syntactic or articulatory processing. Specifically, variation with syntactic complexity would reflect time windows relevant for syntactic encoding during sentence production. Moreover, relevant cognitive functions (i.e., visual memory, attention, working memory) were studied independently using standardized tests and related to the ERP data in order to exclude possible confounding of these more basic functions.

## Materials and Methods

### Ethics statement

The Medical Ethical Committee of Maastricht University Hospital/Maastricht University (azM/UM) gave ethical clearance for this study. All participants, and for minors also both parents/caregivers, gave written informed consent.

### Participants

Twenty-four adolescent patients with galactosemia and twenty-one healthy controls participated in this study. Classic galactosemia was diagnosed by GALT enzyme activity assay and/or GALT-gene mutation analysis. Two participants (both patients) were excluded because of difficulties executing the ERP task. Patient characteristics can be found in Table 1. Of the remaining 22 patients, 15 were female and 7 male, mean age 14.9 years (SD 2.2 y, range 10.8–19.1 y). The control group consisted of 14 females and 7 males mean age 14.2 years (SD 1.8 y, range 11.4–17.0 y). Neither gender nor age differed significantly between the groups [*F*(1,41) = .01, *p* = .92 and *F*(1,41) = 1.07, *p* = .31, respectively]. Participants had no other relevant health conditions, all had normal or corrected to normal vision, and were native Dutch speakers.

**Table 1:** Galactosemia patient characteristics.

	N	Mean	SD	Range of values
**Age at diagnosis (in days)**	22	12.4	14.3	0–60
**Age at introduction of diet (in days)**	22	12.2	14.4	0–60
**GALT activity (in % of mean reference value)** 1 **^,^** 2	20	0.60	0.57	ND^3^ - 1.83
**Urine galactose level (in µmol/mmol creatinine)** 4	22	12.0	21.1	ND^3^ – 96
**Urine galactitol level (in µmol/mmol creatinine)** 4	22	132.0	22.8	94 – 187
**Special education** 5	22	68.2%		
**Speech therapy** 5	22	86.4%		
**Motor therapy** 5	22	50.0%		
**GALT gene mutation**	10	50%	Q188R/Q188R	
	5	25%	Q188R/other^6^	
	5	25%	other^7^	

GALT enzyme activities indicate that all patients have the classic galactosemia type. Urine galactose and galactitol levels indicate adequate dietary compliance.

1 GALT activity was measured at diagnosis;

2 In case the GALT activity is not reported, it was confirmed by the treating physician to be severely decreased;

3 ND = not detected;

4 Urine levels were measured within three months of testing;

5 At some point in life;

6 Q188R/L195P (n = 4) or Q188R/S135W (n = 1);

7 L195P/K229N (n = 3) or 400Tdel/unknown (n = 2).

### Neuropsychological tests

The Rey Osterreith Complex Figure was used to assess visuo-motor skills (Copy subtest), short term visual memory (Immediate Recall) and long term visual memory (Delayed Recall and Recognition) [44]. The Bourdon-Vos test was used to measure sustained attention skills (mean reaction time [RT]) [45]. The Digit Span (Forward and Backward) addressed verbal working memory skills [46].

### Language paradigm during EEG recording

Visually animated scenes were presented to the participants. Each scene consisted of three geometrical shapes (square, triangle, or circle) having one of three different colours (red, blue or green). In each trial, one of the three geometrical figures performed an action upon another figure (one figure moves towards or bumps into another figure; described by either ‘to fly towards’ or ‘to bump into’). Participants were asked to either passively watch the scene (control task, ‘C’) or to describe the animated scene overtly using one of two possible responses that varied in syntactic complexity: using separate words, ‘W’ (e.g., *“triangle”, “red”, “square”, “green”, “to bump into”*; minimal syntactic planning) or using sentences, ‘S’ (e.g., *“The red triangle bumps into the green square.”*; sentence-level syntactic planning) [38], [40]. Participants were asked to keep the naming format of the phrases constant over trials. In the word ‘W’ naming format, lexical access of words is required, but virtually no syntactic encoding. In the sentence ‘S’ naming format, in contrast, syntactic encoding is required on local noun phrase level (e.g., inflection of adjectives) and on sentence level (e.g., inflection of the verb, determination of the word order, constructing and filling in of the syntactic frame). The control (‘C’) condition was added in this study to receive relevant information for the required non-linguistic resources (e.g., visual processes, attention).

### Procedure

The study was conducted in two sessions. In the first session, the neuropsychological tests were conducted in all participants after explanation and written informed consents were given (by the participant and both parents/caregiver). In the second session, the language paradigm and EEG recordings took place. After a brief explanation, participants were prepared and seated in an electrically-shielded, sound-attenuated room in front of a computer monitor. The session started with the control task ‘C’, followed by instructions and a practice version of the language task (consisting of 18 practice trials per condition) and the main language experiment.

**Figure 1:**
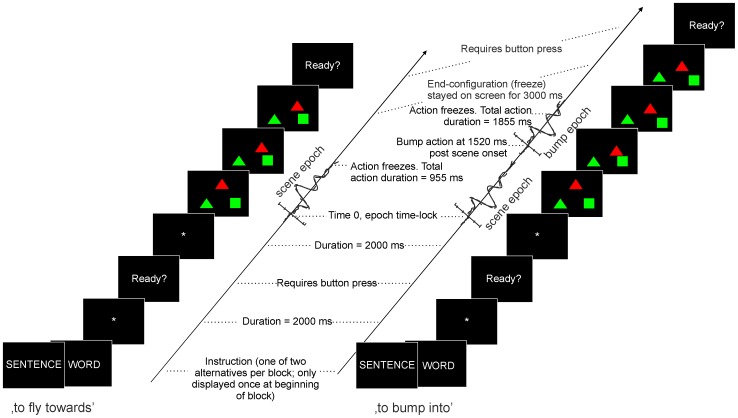
Overview of the sequences of events within trials. Timing of events within an experimental trial, separated for the two action formats (‘to fly towards’ and ‘to bump into’). Time is displayed upwards. The block started with the instruction cue (‘WORD’ or ‘SENTENCE’), a fixation cross, a ready sign, and a randomized sequence of trials. For each trial type screenshots are displayed to illustrate the actual moving time period of the objects along with the moments of expected response of the participant, and the corresponding ERP epochs of interest (time-locked to scene onset and the bump event, respectively).

The main language task consisted of three runs in a blocked design. Each run comprised two blocks which were randomized within the run and counter-balanced between participants to exclude order effects. Each block started with a brief instruction reflecting the expected naming format (i.e., either ‘SENTENCE’ or ‘WORD’) followed by 32 trials of different scene displays, of which the content (figures, colours, action and arrangement) was randomized. Per condition and participant, a total of 96 trials were recorded. The control task consisted of three consecutive runs, having a total of 108 trials. Figure 1 gives a schematic overview of the sequences of events within a trial. The duration of animation in the scene differed (955 or 1885 ms) depending on the action format (‘to fly towards’ or ‘to bump into’, respectively). The difference in animation durations is caused by a different amount of action frames (10 versus 18 frames, where the actual ‘bump’ event occurred at frame 14, at 1520 ms after scene onset). Note that the movements in the scenes are visually identical until they diverge at the moment the ‘to fly towards’ trials freeze while ‘to bump into’ trials continue. Participants were instructed to start the description as fast and accurate as possible. The next trials started via a self-paced button push (USB-keyboard key), except for the control trials which had a fixed 2000 ms interval between trials. Control trials had approximately the same duration as the linguistic trials.

### Electroencephalography (EEG) recording

The EEG recording was done using an elastic cap in which 32 tin electrodes were mounted (Electro-Cap International (ECI), Inc.), positioned according to the international 10–20 system [47]. Twenty electrodes - F3, Fz, F4, FC3, FCz, FC4, C3, Cz, C4, CP3, CPz, CP4, P3, Pz, P4, O1, Oz, O2, T3 and T4 - were measured as active leads, AFz was used as the ground electrode. The left mastoid (A1) was used as online reference. Offline the signal was re-referenced to the average signal of both mastoids. Vertical eye movements and blinks were monitored by two electrodes placed at the left upper and lower orbital ridge. Horizontal eye movements were recorded with electrodes placed on the left and right cantus. The impedance of all electrodes was kept below 5 kΩ. Data acquisition was done using Brain Vision Recorder software (Brain Vision, MedCaT B.V.) and the signal was amplified using a 0.05–50 Hz band pass and sampled at a 500 Hz interval. The scene onset as well as the voice onset triggered a TTL pulse directly into the EEG recordings. The voice onset pulse was initiated whenever the sound pressure level reached a certain threshold (individually adjusted to each subject) and was transferred via a microphone.

### Analyses

The number of errors and self-corrections were computed using the recorded audio data and manual (online) scores. Errors were defined as any deviation from the expected utterance (i.e., incorrect figure, colour, action, naming format or ordering). Self-corrections were defined as any overt corrective effort during the response utterance. The voice onset time (VOT) was determined as the time between the scene onset and the onset of the voice response; the total speech time (TST) was cautiously estimated as the time between the onset of the voice response and the button push indicating when participants were ready to continue. VOTs<0.5 seconds and >4.5 seconds and TSTs<2 seconds and >10 seconds were considered outliers and discarded from analysis. The neuropsychological data were standardized using norm data and classified according to the guidelines of Lezak [48]. A repeated measures General Linear Model was used to analyze the behavioural data (VOT, TST, errors and self-corrections) having Condition (‘W’ versus ‘S’) as the within-subject factor and Group (patients, controls) as between-subject factor. The standardized neuropsychological data were analyzed using frequency tables (for the classified data) and univariate GLM to examine group differences.

With respect to the EEG data, trials in which the participant's response was absent were excluded from analysis. The EEG data were epoched from −200 to 2500 ms post scene onset (to include the entire interval from onset of visual scene to the end of the display/onset of articulation), band-pass filtered from 0.3–30 Hz (zero phase, 24 dB) and baseline corrected (from −200 to 0 ms). Large visual artefacts were removed. In addition, data were decomposed using the infomax Independent Component Analysis (ICA) in EEGlab [49]. This method disentangles brain- and artefact-related processes by searching for maximally independent components [50]. Stereotype artefact-related components reflecting eye movements, noise and muscle activity were subsequently removed. On average, 84.5% of all trials (SD 5.2%) were kept for analysis [no difference between groups, *F*(1, 41) = .000, *p* = .988]: mean 96 trials in ‘C’, 79 in ‘W’ and 78 in ‘S’. The remaining components (the cleaned data) were back-projected into the ERP. In the back-projected ERPs, epochs were divided in two time ranges: one interval related to the scene onset (−200 to 1000 ms after scene onset), and one related to the bump event (−200 to 800 ms after the bump event, or 1320 to 2320 ms post scene onset) (see also Figure 1). Note that in the bump epoch, only ‘to bump into’ trials were included (and no ‘to fly towards’ trials), corresponding to on average 49 trials in ‘C’, 39 in ‘W’ and 40 in ‘S’. The bump epochs were baseline corrected (−200 to 0 ms after the bump event). Based on visual inspection of the grand averages, target peak ERP components and corresponding time windows were specified on which we conducted mean amplitude analyses.

ERP statistics were performed on the mean amplitude data per time window, condition, and participant using repeated measures GLM with Condition as within-subjects factor (‘C’, ‘W’, ‘S’), and two within-subject topographical factors Laterality (left, central, right) and Anterior-Posterior (F, FC, C, CP, P, O). Based on visual inspection, additional analyses were performed on subsets of electrodes. Group was added as the between-subjects factor (patients, controls). Pearson's correlations were used to examine the relationship between the ERP data and behaviour (on-line measures of reaction times and accuracy) and other cognitive functions (off-line neuropsychological tests); and with patient characteristics (e.g., mutation, rest activity of the enzyme). Where necessary, corrections were made for multiple testing (Bonferroni) and for sphericity violations (Greenhouse Geisser). Age and gender were added as covariates in all analyses but the ones performed on standardized data. An alpha of 0.05 was used as significance level.

## Results

### Neuropsychological test results

The patients scored significantly lower compared to controls on the following subtests: Rey Complex Figure Copy, Immediate and Delayed Recall and Recognition; Bourdon-Vos total RT and number of errors; and Digit span [.000<*p*<.027]. However, when the Rey Complex Figure Copy score was subtracted from the Immediate Recall score (not standardized, to control for visuo-motor differences), the groups did not differ [*p* = .75]. Examining the slope of the three Bourdon-Vos RTs (not standardized, to examine the sustainability of attention), the groups did not differ either [*p* = .25]. The groups did not differ on the number of omissions and corrections on the Bourdon-Vos [*p* = .91 and *p* = .33, respectively]. Table 2 gives an overview of the neuropsychological data of the patient group (control data is not presented for clarity reasons).

**Table 2:** Classified neuropsychological data of the patients with galactosemia.

	Very low	Low	Below average	Average	Above average	High	Very high
**Expected distribution**	2.3%	7.4%	17.7%	45.2%	17.7%	7.4%	2.3%
**Rey Complex Figure**							
Copy	68.2%	13.6%	18.2%^1^				
Time to copy	-	8.3%	91.7%^1^				
Immediate Recall	59.1%	22.7%	9.1%	4.5%	4.5%	-	-
Delayed Recall	54.5%	27.3%	9.1%	9.1%	-	-	-
Recognition	27.3%	18.2%	18.2%	31.8%	4.5%	-	-
**Bourdon-Vos**							
Total RT	-	59.1%	27.3%	13.6%	-	-	-
Number of omissions			59.1%	36.4%	4.5%		
Number of corrections			4.5%	45.5%	50.0%		
Number of errors			36.4%	63.3%	-		
**Digit Span**							
	42.9%	23.8%	14.3%	19.0%	-	-	-

Presented are the percentages of patients scoring within the particular classifications as described in Lezak [48]: z<−2 very low; −2<z<−1.3 low; −1.3<z<−0.6 below average; −0.6<z<0.6 average; 0.6<z<1.3 above average; 1.3<z<2 high; z>2 very high. Note that the expected distribution reflects percentages based on the normal distribution.

1 Below average or higher.

### Behavioural data language paradigm

#### Accuracy

**Figure 2:**
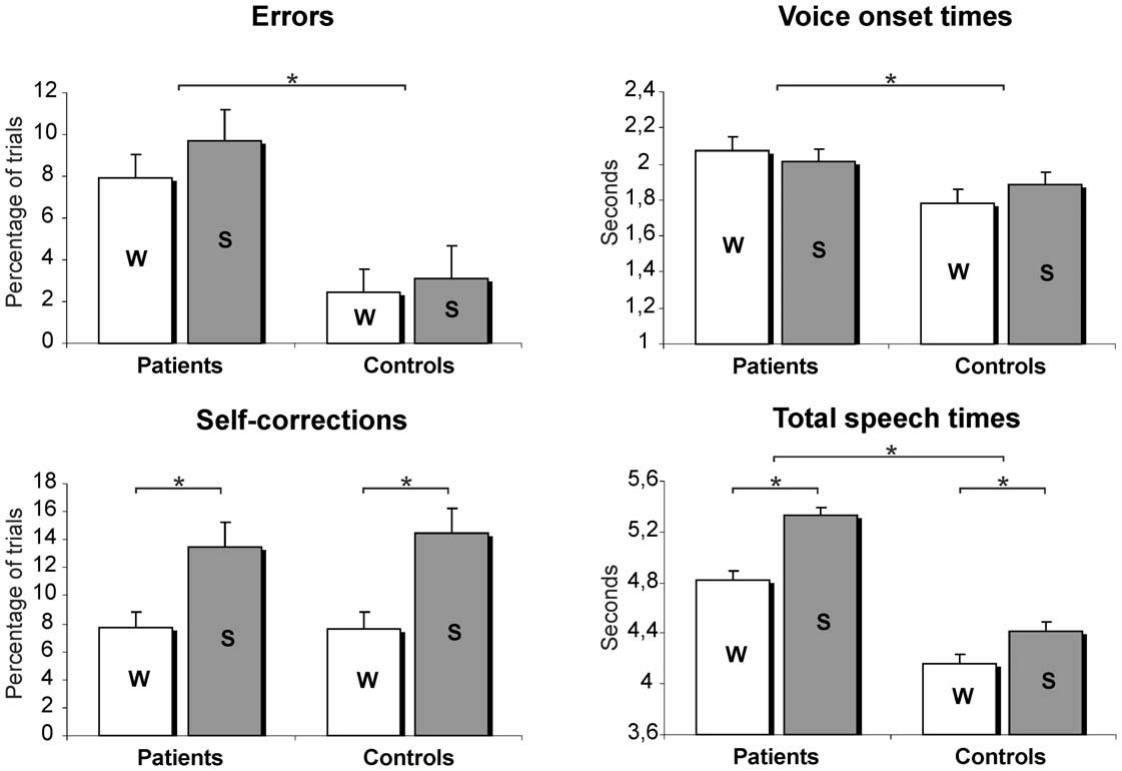
Behavioural data. Behavioural data per group and per condition. ‘W’ = Word condition; ‘S’ = Sentence condition. Presented are estimated marginal means with standard error (SE) bars. Asterisks indicate significant effects (P<.05).

The number of errors differed between groups, [*F*(1,39) = 12.24, *p* = .001]: the patients made more errors than the controls. There was no difference in the number of errors between the word ‘W’ versus the sentence ‘S’ condition [*F*(1,39) = 2.143, *p* = .151]. The number of self-corrections showed no group difference [*F*(1,39) = 0.063, *p* = .801], but a condition effect. More self-corrections were made in ‘S’ compared to ‘W’ [*F*(1,39) = 27.78, *p*<.001] (Figure 2).

#### Reaction times

The patients had longer VOTs and longer TSTs compared to controls [*F*(1,37) = 5.28, *p* = .027 and *F*(1,37) = 13.154, *p* = .001, respectively]. The TST was longer in ‘S’ [*F*(1,37) = 26.406, *p* = <.001]. No condition effect for the VOT was observed in either group [*F*(1,37) = .061, *p* = .807].

#### Correlations behavioural data and neuropsychological data

In both groups, lower scores on the Rey Complex Figure (Immediate and Delayed Recall) were related to more errors [patients: −.543<*r*<−.490, .009<*p*<.021; controls: −.550<*r*<−.478, .010<*p*<.028]. In patients, lower performance on the Rey Immediate recall task was associated with longer TSTs [*r* = −.651, *p* = .001].

### ERP data

**Figure 3:**
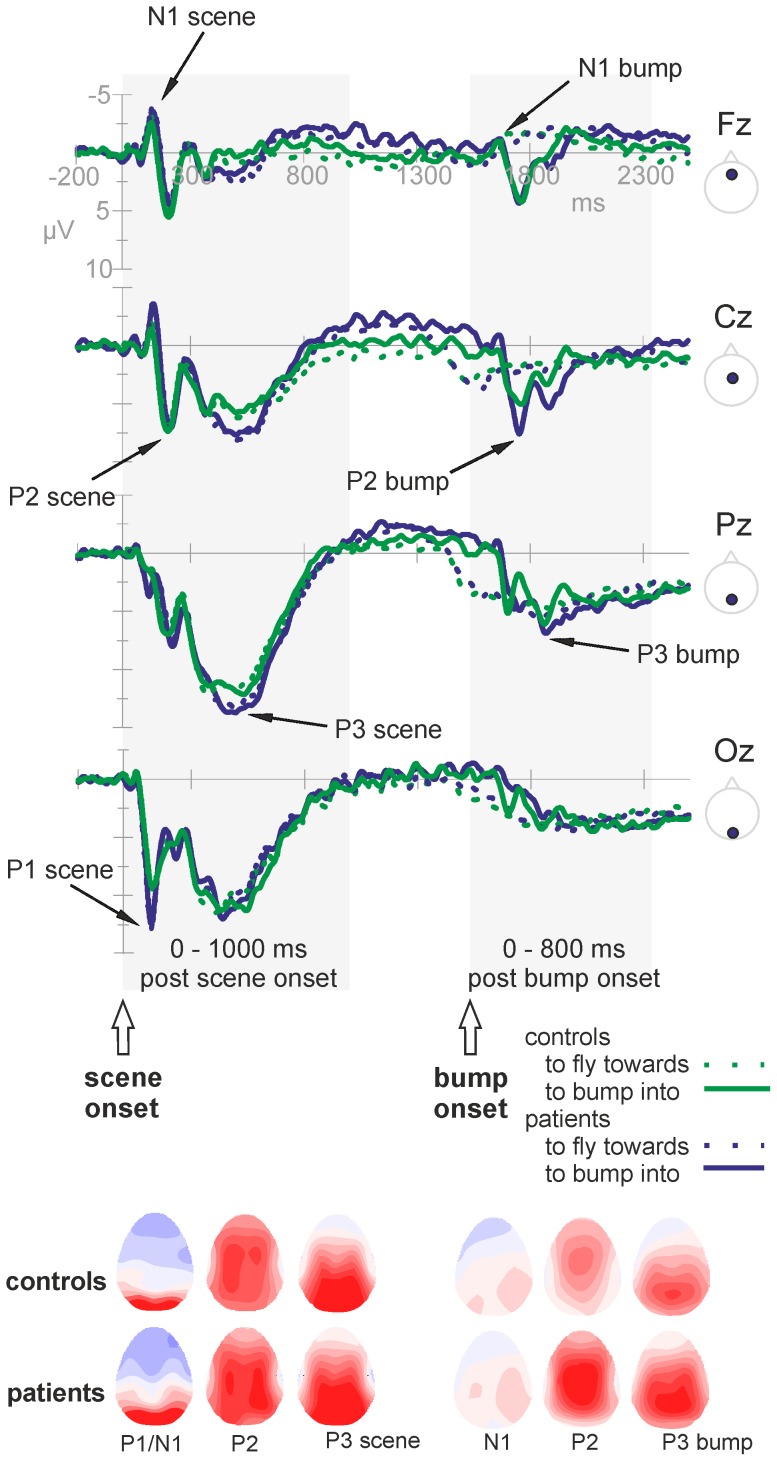
Overview of ERPs and topographies. Top: Grand average ERPs of the patients with galactosemia (blue) and the healthy controls (green) across the midline of the scalp (F = Frontal, C = Central, P = Parietal, O = Occipital). The lines are averaged across conditions, but separate for the two action formats: solid lines represent the ‘to bump into’ format; dashed lines the ‘to fly towards’ format. The two epochs of interest are highlighted: the post scene onset epoch (where scenes of both action formats, and their corresponding ERPs, are still identical) and the post bump event epoch (where the analyses were limited to the ‘to bump into’ trials, as the ‘to fly towards’ trials do not show an ERP morphology during this time window). Negative voltage is plotted up in this and all subsequent figures. Bottom: Overview of the topographical distributions over the scalp of the components of interest, for each group seperately. Both the ERPs and the corresponding topographies illustrate an overall similar morphology for the patients and controls.

The ERP waveforms depict the planning phase of the utterance from scene onset onwards. Figure 3 shows the grand average waveforms of the patients with galactosemia versus the matched controls for the entire epoch interval of −200 to 2500 ms after scene onset (averaged across conditions). Separate lines are shown for the two action formats ‘to fly towards’ and ‘to bump into’. The figure illustrates that the scenes (and the corresponding ERPs) were identical until approximately 1000 ms post scene onset and start to diverge relatively late. Visual inspection of the grand averages showed a clear ERP morphology during the first thousand milliseconds post scene onset, followed by a relatively steady period (in which no event related activity is visible). Another subset of ERP components was observable at a relatively late time interval (from approximately 1500 ms after scene onset onwards), restricted to the bump trials. Analyses were directed towards these two epochs of interest: −200 to 1000 ms after the scene onset (before the action format and thus the verb is available; local syntactic planning) and −200 to 800 ms after the bump event (when the verb is available, corresponding to 1320 to 2320 ms after scene onset, limited to the bump trials; global sentence-level syntactic planning). As the arrows in Figure 3 depict, there are several time points at several electrodes where groups and/or conditions differ, starting early in time. The overall morphology, however, was quite similar (see also topographies in Figure 3). Statistical analyses were carried out across several time windows with labels ‘scene’ referring to components following scene onset, and label ‘bump’ referring to components following the ‘bump’ event: 90–150 ms (referred to as *P1 scene*), 100–160 ms (*N1 scene*), 180–240 ms (*P2 scene*) and 350–650 ms (*P3 scene*) post scene onset; 70–170 ms (*N1 bump*), 180–280 ms (*P2 bump*) and 300–500 ms (*P3 bump*) post bump event. Note that the labels P1, N1, P2 and P3 are used for descriptive purposes.

### Time windows of interest post scene onset

#### Time window 90–150 ms – P1 scene

**Figure 4:**
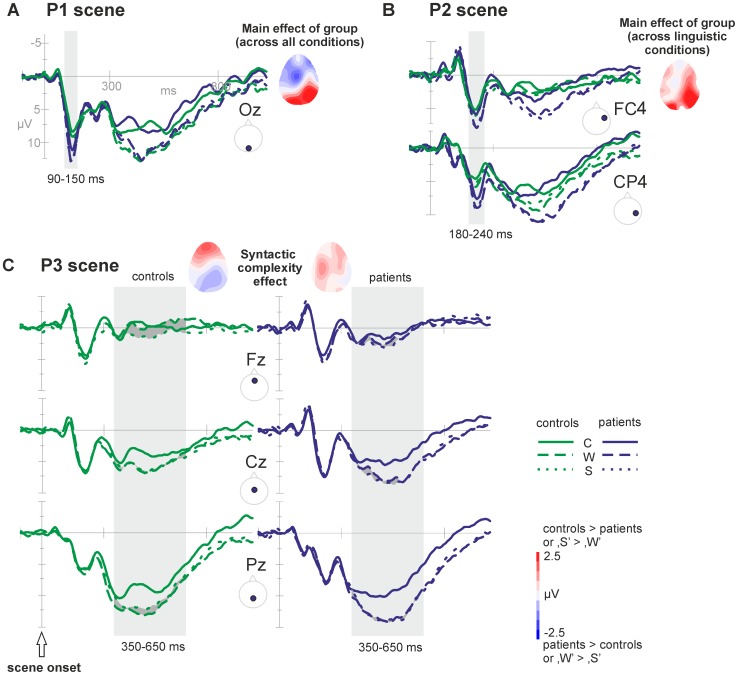
Overview of the ERP effects in the post scene onset epoch. A) Grand average waveforms of the occipital midline electrode (Oz) displaying the P1 Group effect (patients>controls) within the time window 90–150 ms post scene onset. This group effect (difference between groups) is also displayed in the topography. B) Grand average waveforms of two right-hemispheric electrodes (i.e., FC4, CP4) displaying the P2 Group effect (patients>controls; also displayed in the topography) and the lexical access effect (‘W’ = ‘S’>‘C’) within the time window 180–240 ms post stimulus. C) Grand averages of the anterior P3 syntactic complexity effect (‘S’>‘W’; highlighted in dark grey), significant in controls but not in patients, within the time window 350–650 ms post scene onset. The corresponding topographies of the syntactic complexity effect also show the effect in the controls, while no clear effect is observable in the patients.

A positive component was observed with a maximum around 120 ms post scene onset and an occipital scalp distribution. Analyses were restricted to the occipital plane (O). A Group effect [*F*(1, 39) = 6.00, *p* = .019] was visible and significant in all three conditions (‘C’: *p* = .032, ‘W’: *p* = .021, ‘S’: *p* = .021), with the patients' ERP being more positive. The patients but not the controls showed a trend in the Condition effect [*F*(1.59, 30.23) = 2.91, *p* = .081]. Simple contrasts in the patients data revealed that ‘C’ differed from both ‘W’ and ‘S’ [*p* = .004 en *p* = .024, respectively] (see Figure 4A).

#### Time window 100–160 ms – N1 scene

At anterior sites, a negative component was observed at 100–160 ms after scene onset, with a maximum at 130 ms. Analyses were restricted to F3, Fz, F4. No clear condition effect was revealed. The Group effect was not significant either [*F*(1,39) = 3.47, *p* = .070].

#### Time window 180–240 ms – P2 scene

A positive component was observed peaking around 210 ms after scene onset. Analyses showed that this component was largest over midline fronto-central and parietal sites. Because of interactions between Condition and the topographical factors, the analysis was further conducted on sub-regions.

At the right side of the scalp (F4, FC4, C4, CP4, P4) there was a Condition effect [*F*(1.95,76.03) = 7.93, *p* = .001]. Follow-up analyses showed that ‘C’ differed from both ‘W’ and ‘S’ [*p* = .001 and *p* = .007, respectively]. There was no difference between ‘W’ and ‘S’. Recordings at right posterior electrodes (CP4, P4) revealed a Group effect [*F*(1,39) = 4.62, *p* = .038]. Follow-up analyses showed that the patients' ERP signal in the linguistic conditions (but not in passive watching) was more positive compared to controls [‘W’: *F*(1,39) = 4.31, *p* = .044; ‘S’: *F*(1,39) = 4.97, *p* = .032] (see Figure 4B).

Only in controls, better sustainability of attention (lower slope of the Bourdon Vos reaction times) was associated with a larger linguistic condition effect (i.e., difference in mean amplitudes between ‘C’ and both ‘W’ and ‘S’ at FC4) [‘C’-‘W’: *r* = −.444, *p* = .044; ‘C’-‘S’: *r* = −.754, *p*<.001].

#### Time window 350–650 ms – P3 scene

During this time window, a large long-lasting positive activity was observed, with a maximum at posterior sites. Analyses indicated interactions between the Condition effect and the electrode locations. Therefore, further analyses were performed on sub-regions.

In central and parietal regions (FC, C, CP, P), a Condition effect was observed [*F*(1.98,77.34) = 21.19, *p*<.001]. Pair wise comparisons showed that ‘C’ differed from ‘W’ and ‘S’ [both *p*<.001]. The groups did not differ significantly and the Condition effect was the same for both groups. At frontal regions (F), a difference was observed between ‘S’ and ‘W’ [*F*(1,39) = 4.838, *p* = .034]. However, this effect was only present in the controls [*F*(1,18) = 7.589, *p* = .013] and not in the patients [*F*(1,19) = .022, *p* = .884] (see Figure 4C). At the frontal site, there is a trend towards a difference in amplitudes between groups [*F*(1,39) = 2.625, *p* = .113].

In controls, larger syntactic complexity effects (i.e., mean amplitude difference between ‘S’ and ‘W’ at Fz) were associated with shorter TSTs (in ‘S’) [*r* = −.462, *p* = .035]. In patients, longer VOTs (in ‘S’) were associated with smaller syntactic complexity effects [*r* = −.474, *p* = .030].

### Time windows of interest post bump event

#### Time window 70–170 ms – N1 bump

At anterior sites, a negative component was visible at 70–170 ms after the bump event, on average peaking at 130 ms. Analyses were restricted to frontal and fronto-central electrodes (F FC). There was no significant Condition effect [*F*(1.83,71.36) = 1.96, *p* = .151], nor any interaction effects. The groups did not differ either [*F*(1,39) = 0.65, *p* = .43].

#### Time window 180–280 ms – P2 bump

Around 230 ms post bump event, a large positive component was observed. The topographic distribution was fronto-central. Analyses therefore were restricted to these electrodes (F FC C). In addition to Condition and Group effects, interaction effects were found between the factor Anterior-Posterior and both Group and Condition.

**Figure 5:**
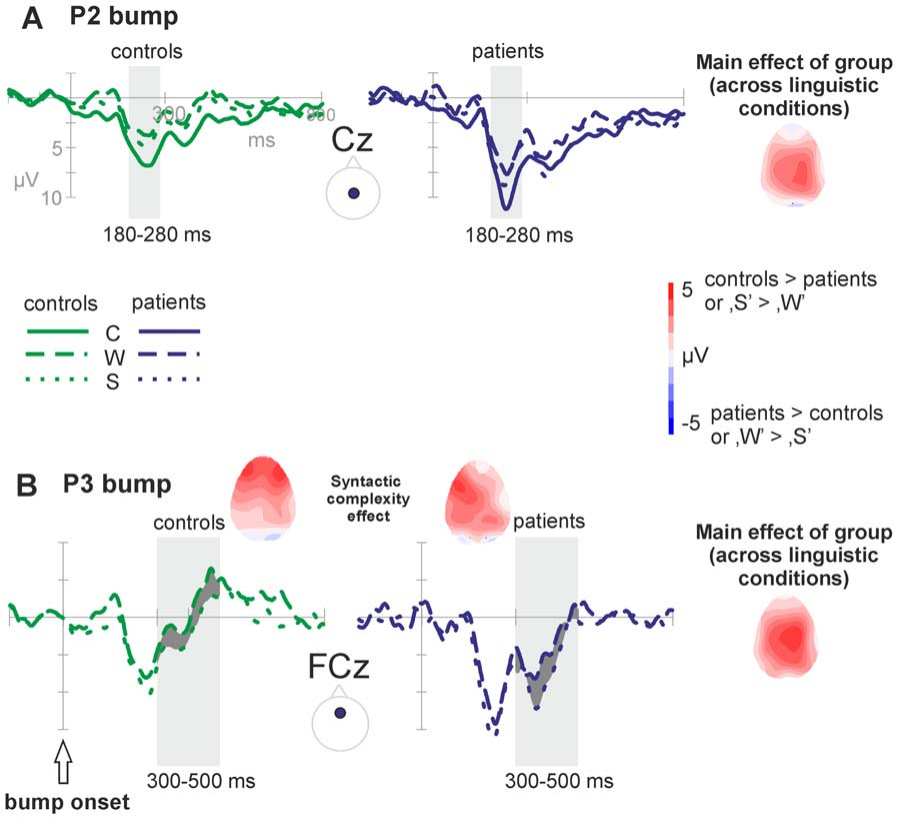
Overview of the ERP effects in the post bump event epoch. A) Grand average waveforms of the central midline electrode (Cz) displaying the P2 bump group effect (patients>controls; also reflected in the topography) and the P2 bump linguistic condition or lexical access effect (‘W’ = ‘S’<‘C’) within the time window 180–280 ms post bump event. B) Grand averages of the fronto-central midline electrode (FCz) reflecting the P3 bump syntactic complexity effect (‘S’>‘W’; reflected in the dark grey fill in the ERPs and in the corresponding topographies) and the P3 group effect (patients>controls; see also topography) within time window 300–500 ms post bump event.

Sub-analyses revealed only a marginal Condition effect in the FC plane [*F*(2.0,77.91) = 2.94, *p* = .059], while the Group effect was significant (*F*(1,39) = 9.42, *p* = .004]. In the central plane (C), there was a clear Condition effect [*F*(1.99,77.71) = 9.24, *p*<.001], where ‘C’ differed from both ‘W’ and ‘S’ [*p* = .000 and *p* = .006, respectively]. The two linguistic conditions did not differ [*p* = .214]. The Group effect was significant [*F*(1,39) = 12.88, *p* = .001], reflected in more positive amplitudes in the patients' ERP compared to controls (see Figure 5A).

Only in controls, larger linguistic condition effects (i.e., difference between non-linguistic and linguistic conditions at Cz) were associated with shorter VOTs [‘C’-‘W’ effect: *r* = −.497, *p* = .033; ‘C’-‘S’ effect: *r* = −.633, *p* = .002] and with fewer errors [‘C’-‘W’ effect: *r* = −.517, *p* = .016; ‘C’-‘S’ effect: *r* = −.602, *p* = .004]. Only in patients, better visual memory performance (Rey Complex Figure Immediate and Delayed Recall) was related to larger linguistic condition effects (‘C’-‘W’) [*r = *.570, *p* = .006; *r* = .611, *p* = .003, respectively].

#### Time window 300–500 ms – P3 bump

Between 300 and 500 ms post bump event, a large positive component was observed. The component was broadly distributed, with a maximum over posterior sites (CP P). Analyses were performed on F FC C CP P electrode lines. In addition to a significant Condition effect, there were interactions between the Anterior-Posterior topographical factor and the Condition factor. The groups differed across the entire scalp [*F*(1,39) = 11.21, *p* = .002; in all conditions, ‘C’: *p* = .046, ‘W’: *p* = .010, ‘S’ *p* = .001].

Sub-analyses revealed a Condition effect that was present in all planes (except for F), but was largest at posterior sites [P: Condition *F*(1.95,76.03) = 9.08, *p*<.001]. Follow up analyses showed that ‘C’ differed from both ‘W’ and ‘S’ [*p*<.001 and *p* = .002, respectively]. Posterior, the linguistic conditions did not deviate. Anterior, however, ‘W’ and ‘S’ differed significantly [FC: *p* = .025], with ‘S’ being more positive than ‘W’. There was no interaction between Group and Condition (see Figure 5B).

Only in controls, better sustainability of attention was associated with larger syntactic complexity effects (i.e., difference in mean amplitude between ‘S’ and ‘W’ at FCz) [*r* = .498, *p* = .022].

### Associations between outcome data and patient characteristics

There were no significant correlations between the patient characteristics (i.e., age at introduction diet, GALT enzyme activity, urine galactose and galactitol values) and the ERP data. Inspection of correlations with behavioural data revealed that older age at introduction of diet was related to longer TSTs [*r* = .689, *p* = .001]. Further, higher urine galactitol values were correlated with the shorter VOTs [*r* = −.514, *p* = .017]. Better verbal working memory scores (Digit Span) were related to lower galactitol values in urine [*r* = −.471, *p* = 0.031].

Differential effects for patients with different genotypes were explored (homozygous for Q188R versus other mutations). The GALT enzyme activity and urine galactose and galactitol values did not differ across groups. The Q188R homozygous group had longer VOTs [only in the ‘W’ condition, *F*(1,18) = 5.213, *p* = .036]. No differences in neuropsychological scores were found, but the groups differed with respect to the ERP effects: the syntactic complexity effect (i.e., difference between the linguistic conditions in the P3 bump time window) was greater in the ‘other mutation’ group compared to the homozygous group [*F*(1,19) = 13.362, *p* = .002].

## Discussion

This study is the first to apply theories, methods, and experimental paradigms from cognitive neuroscience to study language production impairments in classic galactosemia. This approach reveals impairments in several language production stages prior to articulation in these patients.

### Behavioural data

The adolescent participants described animated scenes using different syntactic complexity formats: either separate words (‘W’) or complete sentences (‘S’). Both groups required more self-corrections and speaking time in the sentence condition as compared to the word condition, suggesting that the intended complexity variation of syntactic planning was successful. Several outcome measures are found to deviate in patients compared to matched controls. The patients made more errors than controls (8.8% versus 2.8% of all trials). They needed more time to prepare (VOT 2.0 versus 1.8 seconds) and to finish the utterance (TST averaged across conditions: 5.1 versus 4.3 seconds), indicating that the patients were both slower and less accurate. Interestingly, in both groups, the error rates and voice onset times did not differ across the sentence and the word condition. The finding that the speaking time is modulated by syntactic complexity, but the voice onset time is not, suggests that most of the syntactic planning occurs after the initiation of the utterance.

### ERP components of healthy controls

The ERPs reflect the entire information processing sequence, including visual processing of the figures and their movements, and the language planning process. We will first discuss the effects of the condition modulations in the control group only, in order to make inferences on their functional relevance. Several time windows showed a condition modulation, before and after the action format (the verb) became clear, reflecting the early initiation of the utterance and the incremental nature of the language planning.

The ***P1 scene*** is, with respect to distribution and latency, most likely an instance of the occipital P100, traditionally associated with visual and attention processes [51], [52]. The P1 has also been linked to motion processing of visual stimuli (i.e., influenced by on- and offset, linked to V1) [53] and to conceptual processes [21], [54]. There was no modulation with condition, indicating similar requirements for motion processing, attention and conceptualisation across the conditions in this study. The ***P2 scene*** component is most likely a P200, traditionally observed over anterior sites [52] and linked to lexical access of words during picture naming [55], [56] or word reading [57]. Along this line, the observed effect likely reflects lexical access, as the linguistic conditions (‘W’ and ‘S’) do not differ in lexical requirements or P2 modulation, but differ from passive viewing (not requiring lexical access). Although the scene just started at this point, it is already clear which figure is the actor and which object is involved in the action, while the action format - verb - is still ambiguous. Therefore, lexical access is most likely restricted to access of the first noun phrase (actor). The idea that planning starts with the onset of the visual stimuli is consistent with the idea that language production is (at least partly) driven by visual input or visual attention [58]–[60]. The long lasting and widely distributed ***P3 scene*** resembles a P300. Anterior, the P3 showed variation with syntactic complexity. The timing and direction of the effect is in line with previous reports of the P300 reflecting integration of working memory and attention, both necessary for updating incoming information over time [61]. The observed ERP variation with syntactic complexity at the frontal midline (where ‘S’ is more positive than ‘W’) can be explained by the need for more attention- and memory-related resources in case of higher syntactic complexity, or could be a direct indication for more complex syntactic processing. At this time point, the action format (verb) is still ambiguous as differentiation between the two potential verbs can only happen after appropriate visual input (the bump, at 1520 ms after scene onset). Based on this, we conclude that syntactic planning reflected by the P3 must be restricted to local syntactic processing (i.e., retrieval of syntactic information about the actor/noun, inflection of the adjective). As there was no syntactic complexity effect in the VOT, we can assume that the utterance is initiated prior to syntactic planning once the first element of lexical access is in (noun or noun phrase) [32], [33]. Larger P3 syntactic complexity effects were associated with shorter TSTs, indicating that more advanced local syntactic planning decreases the speaking time or increases the efficiency of the language process. In the following time window, the ERP shows activity around baseline (approximately 900 to 1400 ms post scene onset), presumably reflecting neural activity without clearly measurable events (eventually due to high variation in cognitive processing within and between groups). Then, divergence across action formats (verbs) occurs both scene-wise and ERP-wise. Time-locked to the moment of the bump, another set of ERP components arise (in the ‘to bump into’ trials only, presumably because the lack of a clear temporal event in the ‘to fly towards’ trials). During the fronto-central ***P2 bump*** component, we observed a condition pattern identical to that of the *P2 scene* component: the two linguistic conditions differed from passive watching, but not from each other. Now, all information is available (including the verb), making lexical access of the verb possible in an unambiguous way. Larger linguistic condition effects (i.e., difference between non-linguistic and linguistic conditions) were related to shorter VOTs and less errors, indicating that larger linguistic condition effects are associated with more accurate and faster performance. Finally, the large and widely distributed ***P3 bump*** component probably reflects a P300. Again, this post-bump P3 showed a similar pattern as the post-scene P3: variation with syntactic complexity. At this point, not only local but also global syntactic planning is required in the sentence condition (i.e., combination and integration of all noun phrases and the verb into a well-formed sentence), reflected in the larger P3 amplitudes. To sum, the functional interpretation of the ERPs in healthy controls is such that it starts with a set of components related to processing of moving visual information/conceptualisation (*P1 scene*), lexical access of the noun phrases (*P2 scene*) and local syntactic planning of the noun phrases (*P3 scene*). When all information, including the verb, is available, the ERP continues with similar components related to lexical access of the verb (*P2 bump*) and to syntactic planning on a more global sentence-level (*P3 bump*).

Relatively few studies have examined overt naming during ERPs recording, especially not using multi-word utterances [22], [55], [62], [63]. Marek et al. [62] elicited multi-word utterances and sentences and found a posterior P3-like component (350–500 ms post stimulus) reflecting syntactic complexity (in addition to increasing conceptual complexity, as the used paradigm did not disentangle the two). In the present study, conceptual complexity was kept constant, suggesting that the observed P3 modulations speak to syntactic complexity proper. The production P3's that are found in the current study and the described previous studies, might therefore be analogous to the P600/SPS in syntactic comprehension [37], [64], [65], albeit with a more anterior distribution of the syntactic effect. The finding that both instances of the P3 in this study (post scene and post bump event) display the syntactic complexity effect provides additional support for a role for syntactic encoding in this component.

Psycholinguistic models of speech processing assume incremental planning of an utterance [15], [17], [35]. In our study, the utterance seems to be initiated after lexical access of the first noun, but prior to syntactic planning of this noun phrase (as the VOT did not vary with syntactic complexity). Our results therefore support the idea that an utterance can be initiated before the visual stimulation is finished (and before all necessary information is available). In addition, by means of ERP variations, we could look into the linguistic planning phase. Well before voice onset and before the visual input of the scene is complete, we observed activation related to local syntactic planning. When all information was available, there was continuation of syntactic encoding (on a more global, sentence-level). Although this paradigm was originally implemented using PET [38], [40], this study demonstrates its suitability for high temporal resolution methods, since it allows us to disentangle this early local and later more global planning.

### ERP components of patients with galactosemia

The patient ERPs showed a similar morphology compared to that of the matched controls, suggesting a generally intact neural network of cognition and language processing. The patient ERP differed from those of controls in several time windows. In the ***P1 scene*** component, related to attention, visual integration of moving objects and conceptualisation processes, the patients showed higher amplitudes in all three conditions (classically interpreted as more effortful processing) compared to controls. The fact that the patients differ in all conditions from controls, including passive watching, suggests early visual or attention processing deficits or an increased effort to integrate moving objects over time. Moreover, the patients showed a difference between linguistic and non-linguistic conditions (‘W’/‘S’ versus ‘C’) that was not present in controls, suggesting linguistic effects in this early time window, likely reflecting impaired conceptualization. This is the first evidence that the patients diverge at an early stage in cognitive information processing from healthy controls during the preparation of language. In the ***P2 scene***, associated with lexical access, the patients showed the same pattern of condition effects as the controls (difference between control condition and both language conditions). Posterior, the patients showed greater amplitudes in both language-related conditions compared to controls, suggesting difficulties with lexical access. During the ***P3 scene***, the patients did not show the syntactic effect. The finding that the controls showed this syntactic variation but the patients did not can be interpreted as a ceiling effect for the patients: the sentence condition does not diverge from the word condition, as the ceiling level of memory/attention resources is already reached in the word condition (descriptively corroborated by the grand averages showing that in the patient ERP both the ‘W’ and ‘S’ condition are in the same range as the ‘S’ condition in controls). It could be that the patients perform less efficient advance syntactic planning. In controls, larger syntactic complexity effects (i.e., more advance syntactic planning) were associated with shorter TSTs. The patients needed more speaking time compared to controls, also indicative of less (efficient) advance syntactic planning. Different from the controls, smaller syntactic complexity effects were related to longer VOTs, suggesting that for the patients, less advance syntactic planning is related to slower or later initiation of the utterance. The patient' ERP further deviates from controls in the ***P2 bump*** component, providing additional support for impaired lexical access in the patients. Finally, the groups differed from each other during the ***P3 bump*** component, with the patients having larger mean amplitudes compared to controls. The syntactic variation was also present in the patients (opposite to the *P3 scene* component, where only the controls showed this variation). Two explanations are: they require more resources when engaging sentence-level syntactic planning (explaining the higher amplitudes) or, they compensate for earlier impairments in local syntactic planning by engaging in both local and sentence-level syntactic planning at a later (post bump) planning phase causing the higher amplitudes. We cannot disentangle between these alternatives at the moment.

The finding that there were no significant (or minor) differences in the ERP morphology between the groups in passive watching confirms an overall comparable cognitive system, suggesting that behavioural language impairments of the patients are not part of a severe general impairment. This assumption receives empirical support by the observed difference in ERPs between groups for the linguistic task. These differences cannot be explained merely by differences in the visual processing between conditions, but must be related to higher language function – as this was the task manipulation. Besides language planning the effects could be explained by variation in attentional or memory resources. Such variation across different naming formats cannot be excluded.

We investigated whether the observed impairments were purely linguistic in nature or whether they can be explained by other cognitive difficulties by looking into their neuropsychological test profile and by comparing the ERP with test results of specific cognitive functions. As reported in the result section and consistent with previous reports [5]–[8], the patients scored lower on several neuropsychological tests. The patients were slower (Bourdon-Vos reaction times [45]) and had difficulties with the visuo-motor task (Rey Complex Figure Copy subtest [44], among other things requiring the integration of a multitude of components into a unifying whole). Important as well is that visual working memory, when corrected for the visuo-motor differences, was not significantly worse in the patients. Therefore, visual working memory (keeping the visual scene online and actively in mind) cannot explain the behavioural and ERP-related differences between the groups. Verbal working memory performance [46], however, was lower in the patients, potentially adding to the language impairments. Importantly, verbal working memory scores were not correlated to the behavioural and ERP effects during the language task. The lack of correlation suggested that verbal working memory did not directly contribute to the observed ERP effects. Interestingly, several domains that are affected in the patients with classic galactosemia (i.e., visuo-motor skills, motion processing) require some form of integration of information over time. Such an integration deficit may also lead to the difficulties in constructing syntactic frames as well as difficulties to access and fill in the right words into these frames [18].

Correlations with patient variables (i.e., GALT enzyme activity, age at introduction diet, urine galactose and galactitol values) were far from robust, consistent with previous studies failing to find predictive value for these variables [6], [31], [66]. We observed that patients homozygous for the Q188R mutation performed worse on certain aspects compared to patients with other mutations, which is in line with other, but not all, studies [66]. Patients with the Q188R/Q188R mutation had longer VOTs and showed smaller syntactic complexity effects in the P3 bump ERP component.

Previous studies in classic galactosemia have reported general cognitive slowing and diffuse white matter abnormalities [4], [30], [67], theoretically linked to deficient galactosylation of cerebrosides (an important component of myelin) [68]. In line with these findings, our study showed longer reaction times for the patients (both the time needed to prepare and to finish the utterance). In the ERP data, we did not find any delays in the overall evolution of the ERP components. The morphology of the signal was similar for patients and controls. We observed amplitude differences, suggesting an alteration in the neural activity related to a certain cognitive processing phase, which indicates that brain abnormalities might be more clustered than previously suggested. Within the *P3 scene* time window we see a comparable onset of the component, but the P3 seems to be extended in time for patients compared to matched controls. As depicted in Figure 4C, for controls the ERP signal for the ‘W’ and ‘S’ conditions catches up sooner with the signal of the ‘C’ condition, especially more posterior. This overall ERP pattern of the patients suggests that the local neural circuits work within time windows that are comparable to those of healthy controls. However, the larger amplitudes in the patient ERPs indicate aberrant neural activation patterns. Accumulating metabolites or resulting deficiencies that alter neuronal signalling might be involved herein (e.g., myo-inositol [69]). In contrast, the overall integration problem might result from problems of long distance neural communication possibly associated with myelin abnormalities compromising information transfer [30], [67]. However, whether abnormal cell signalling and/or brain connectivity is affected and in which specific regions requires further investigation.

To summarize, patients with classic galactosemia show difficulties in this language production task, both behaviourally (less accurate and slower) and in their ERPs, compared to healthy controls. The ERP deviations start already around the time that attention is directed towards the relevant moving objects and conceptual knowledge of these objects becomes available, suggesting that these processes are affected by the disease. The ERP differences continue throughout the consecutive linguistic preparation phases, indicating affected lexical access and impaired syntactic planning (both local and sentence-level syntactic planning). We conclude that, although anecdotal reports have appeared on weak word retrieval and sentence construction, this study is the first to provide neuro-cognitive evidence for language impairments in patients with classic galactosemia. These impairments affect the planning of language, which occurs prior to the output stage. Based on the ERP data, we suggest that these impairments are related to problems in lexical access and syntactic planning of an utterance. These findings are relevant for speech and language therapies within this patient group, deserving further investigation.
